# Probabilistic Model-Based Cell Tracking

**DOI:** 10.1155/IJBI/2006/12186

**Published:** 2006-07-02

**Authors:** Nezamoddin N. Kachouie, Paul Fieguth, John Ramunas, Eric Jervis

**Affiliations:** ^1^ Department of Systems Design Engineering, University of Waterloo, Waterloo, Ontario N2L 3G1, Canada; ^2^ Department of Chemical Engineering, University of Waterloo, Waterloo, Ontario N2L 3G1, Canada

## Abstract

The study of cell behavior is of crucial importance in drug and disease research. The fields of bioinformatics and biotechnology rely on the collection, processing, and analysis of huge numbers of biocellular images, including cell features such as cell size, shape, and motility. However manual methods of inferring these values are so onerous that automated methods of cell tracking and segmentation are in high demand. In this paper, a novel model-based cell tracker is designed to locate and track individual cells. The proposed cell tracker has been successfully applied to track hematopoietic stem cells (HSCs) based on identified cell locations and probabilistic data association.


Hindawi Publishing Corporation
International Journal of Biomedical Imaging
Volume 2006, Article ID 12186, Pages 1–10
DOI 10.1155/IJBI/2006/12186

### Probabilistic Model-Based Cell Tracking

**Nezamoddin N. Kachouie,[1] Paul Fieguth,[1] John Ramunas,[2] and Eric Jervis[2]**

[1] *Department of Systems Design Engineering, University of Waterloo, Waterloo, Ontario, Canada N2L 3G1*
[2] *Department of Chemical Engineering, University of Waterloo, Waterloo, Ontario, Canada N2L 3G1*

Received 3 February 2006; Revised 28 April 2006; Accepted 12 May 2006

The study of cell behavior is of crucial importance in drug and disease research. The fields of bioinformatics and biotechnology rely on the collection, processing, and analysis of huge numbers of biocellular images, including cell features such as cell size, shape, and motility. However manual methods of inferring these values are so onerous that automated methods of cell tracking and segmentation are in high demand. In this paper, a novel model-based cell tracker is designed to locate and track individual cells. The proposed cell tracker has been successfully applied to track hematopoietic stem cells (HSCs) based on identified cell locations and probabilistic data association.

Copyright © 2006 Nezamoddin N. Kachouie et al. This is an open access article distributed under the Creative Commons Attribution License, which permits unrestricted use, distribution, and reproduction in any medium, provided the original work is properly cited.

### 1. INTRODUCTION

Recent advances in cell culture and cell imaging have made possible the automated acquisition of millions of cell images. The corresponding automation of the analysis of such huge sets of images would allow fundamentally new questions to be addressed in proteomics, genomics, and stem-cell research [1–8]. This paper proposes a coupling of advanced methods in pattern recognition and image processing [9–16] to an existing cell-imaging platform [17] in which the analysis, currently being undertaken by hand, is impossibly slow and tedious for the volumes of data being generated.

The object of this particular project is the analysis of stem-cell behavior and differentiation, the process by which stem cells specialize to different cell types, a process which is crucial to understand if stem cells are to be used in cell and tissue regeneration. Specifically, given a culture of cells, observed over time, we need some way of determining whether a given cell is likely to die, to cause cancer, to specialize into an incorrect tissue type, or, desirably, to specialize into the correct cell type.

The first major step in this process, and the end goal of the research described in this paper, is the automated construction of cell lineage trees, essentially the descendent family tree of a single ancestor cell, as illustrated in Figure 1. Building such a tree for each of multiple cells in a culture requires maintaining cell identity over time, clearly requiring the tracking and associating of cells over a long sequence of images, typically 7000 images taken over a period of several days.

### 2. PROBLEM FORMULATION

Although cell tracking is among the most important and common tasks for biomedical researchers it continues to be undertaken manually. Researchers visually perform cell motion analyses and observe cell movement or changes in cell shape for hours to discover when, where, and how fast a given cell moves, divides, or dies. This task is tedious due to the often corrupted or blurred images, the presence of clutter, the fixing of eyes for long periods of time, and repeating the same task for different cell types. Furthermore, with imaging data ever more simply and rapidly acquired, manual tracking becomes progressively impractical. As a result, automated cell tracking systems are mandatory to further advance the study of biological cells [2, 3, 6, 8, 18–20].

To produce the data for this study, HSC samples are first extracted from mouse bone marrow, then cultured in custom arrays having up to forty wells. A small fraction of a typical HSC microscopic image is depicted in Figure 2 with the superimposed dynamics of a mature blood stem cell before and after splitting. The cells were imaged using manual focusing through a 5X phase contrast objective using a digital camera (Sony XCD-900) and acquired by an IEEE 1394 standard (FireWire) connector. Images were sampled every three minutes over the course of several days.

To keep cells alive and healthy, light exposure must be controlled during their life cycle to minimize phototoxicity. Therefore it is desired to limit light exposure in each frame and to sample the frames as far apart as possible, leading to infrequent, poorly contrasted images, directly at odds with

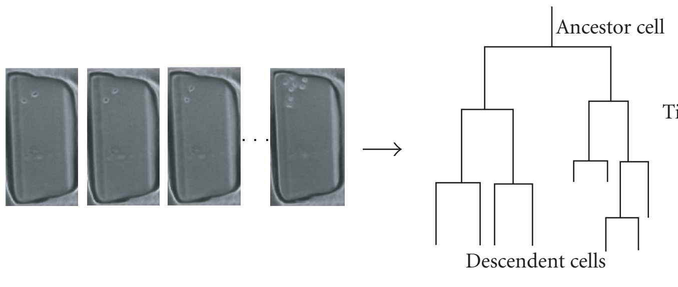

FIGURE 1: An image sequence (a) showing cell (small light circles) movement and division over time. A lineage tree (b) may be generated by detecting cell splitting and associating individual cells from image to image.

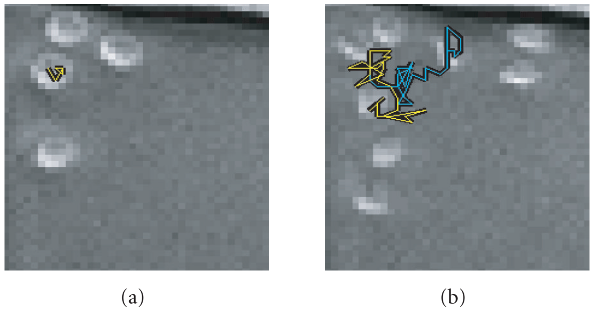

FIGURE 2: Close-up of an HSC phase contrast microscopic image with the superimposed track of one mature blood stem cell (a) 8 frames before and (b) 30 frames after splitting.

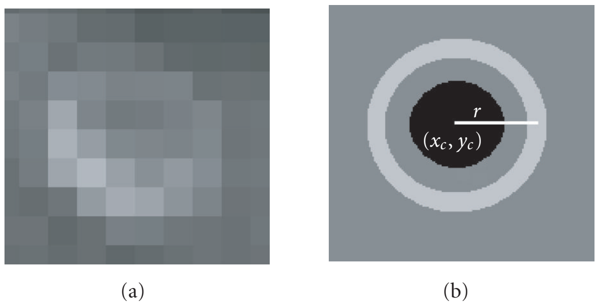

FIGURE 3: (a) 8 by 8 pixel detail of an HSC phase contrast microscope image. (b) A circular idealized cell model.

the data desired for easy tracking: frequent, high-contrast images. Cell staining techniques may be used to increase the contrast between cell and background, however different parts of tissue are undesirably stained unevenly, causing inhomogeneity. Fortunately the HSCs in our study have fairly regular shape and brightness patterns. Hence, a segmentation method which exploits these attributes should be able to perform better than simple thresholding.

Suppose we have an image sequence $I_{1:K} = \{I_1, I_2, \ldots, I_K\}$. The two fundamental tasks needed to construct a lineage tree, such as in Figure 1, are the detection of cells in each image and the subsequent association of the detected cells over time. The cell detection problem is essentially one of anomaly detection: the localization of groups of pixels inconsistent with the random behavior of the image background. A wide variety of semi automatic or automatic methods have been proposed to segment cell boundaries [2, 3] which can be divided into three major categories.

(1) Boundary based, generally employing deformable models such as snakes [7].
(2) Region based, such as split and merge [21], morphological operators [22], watershed [6], and region growing methods [23].
(3) Threshold based [8, 24, 25] applied to some extracted image feature.

We can make the problem much more specific by seeking particular features consistent with HSCs. From Figures 2 and 3(a), HSCs can be characterized as an approximately circular object with a darker interior and a brighter boundary—an effect due to phase contrast imaging modality. So rather than a heuristic thresholding approach, these cell attributes allow a more specific model, essentially a matched filter, more robust to noise and to low contrast. The model depicted in Figure 3(b) considers the following criteria:

(i) cell size: the radius $r$ is known to lie in a limited range related to cell age;
(ii) boundary brightness: brighter due to phase contrast imaging;
(iii) interior brightness: tends to be dimmer than the boundary;
(iv) boundary uniformity or symmetry: want to assert uniformity to avoid a strong response when straddling cells, as shown in Figure 4.

As depicted in Figure 3(b), to model a dark region surrounded by a bright boundary, the proposed cell model consists of two concentric circles, with the radius of the internal circle being half that of the external one. To facilitate the analysis of the image as a function of cell center location $(x_c, y_c)$ and radius $r$ we construct the set of boundary pixels

$$B(x_c, y_c, r, I) = \left\{ I_{ij} \mid \left| (x_c - i)^2 + (y_c - j)^2 - r^2 \right| \leq \left(\frac{1}{2}\right)^2 \right\}, \tag{1}$$

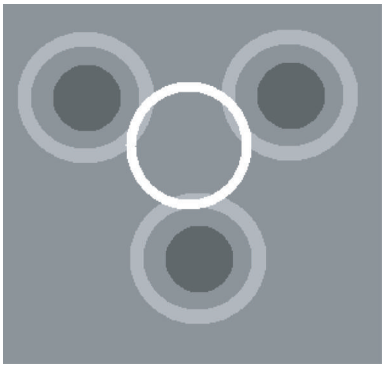

Figure 4: A scenario in which a spuriously hypothesized (white) boundary may have a large associated average brightness $\overline{B}$ and a low cell interior brightness $\overline{C}$. The uniformity constraint in the cell boundary is intended to address this case.

and the set of interior cell pixels

$$C(x_c, y_c, r, I) = \left\{ I_{ij} \mid (x_c - i)^2 + (y_c - j)^2 \le \left(\frac{r}{2}\right)^2 \right\}, \tag{2}$$

from which we extract sample means

$$\overline{B} = \frac{\sum_i B_i}{|B|}, \qquad \overline{C} = \frac{\sum_i C_i}{|C|}, \tag{3}$$

where $B_i$ or $C_i$ is the $i$th element of the respective set.

The four cell criteria are then combined to formulate the following probabilistic cell model:

$$P(x_c, y_c, r \mid I_k) = P_{cb}(\overline{B}) \cdot P_{ci}(\overline{C}) \cdot P_{bu}(B), \tag{4}$$

where the cell boundary $P_{cb}$, cell interior $P_{ci}$, and boundary uniformity $P_{bu}$ terms are elaborated below.

Based on a visual examination of the distribution of sample points of $\overline{B}$ derived from real imagery, the probability density of cell boundary $P_{cb}$ is modeled as Gaussian with mean $\mu_{cb}$ and variance $\sigma_{cb}^2$

$$P_{cb}(\overline{B}) \sim N(\overline{B}; \mu_{cb}, \sigma_{cb}^2), \tag{5}$$

where $\mu_{cb}$ and $\sigma_{cb}^2$ are estimated empirically.

Similarly the probability density of dark region inside the cell $P_{ci}$ is also modeled as Gaussian with mean $\mu_{ci}$ and variance $\sigma_{ci}^2$

$$P_{ci}(\overline{C}) \sim N(\overline{C}; \mu_{ci}, \sigma_{ci}^2), \tag{6}$$

where $\mu_{ci}$ and $\sigma_{ci}^2$ are again estimated empirically.

It should be mentioned that the parameters of model (4) are time invariant, consistent with most of our acquired data sets. Therefore in cases where the intensity or contrast of the image changes over time due to background noise or spatiotemporal illumination variations, the nonstationarity of the data might make (4) in error or inapplicable. In such cases, to improve the robustness of the proposed method, the time variations of the image sequence need to be removed by background estimation and subtraction, considered in future work.

As illustrated in Figure 4 we wish to penalize spurious cell detection. We propose to calculate an empirical cumulative density function (CDF) to discriminate background from cell boundary. The CDF on cell boundary pixel intensities is computed by

$$\mathrm{cdf}_n(B) = \frac{\sum_{i=1}^{n} B_i}{|B| \cdot \overline{B}}, \quad n \in 1 : |B|. \tag{7}$$

As a set of constant or uniform values in $B$ corresponds to a straight line CDF, we use a Kolmogorov-Smirnov test on $B$ to test its deviation from uniformity:

$$D(\mathrm{cdf}) = \max_{n \in [1:N]} \left| \mathrm{cdf}_n - \frac{n}{N} \right|. \tag{8}$$

An exponential function $P_{bu}(D)$ is used to penalize the nonuniformity as

$$P_{bu}(D) = \exp\{ -2 \cdot N \cdot D(\mathrm{cdf}) \}. \tag{9}$$

This completes the development of a simple model for the detection of cells in background noise. We will use the model to generate and test cell hypotheses in the following section.

### 3. CELL TRACKING

With a model in place describing the spatial pattern of pixels with the appearance of a cell, we move to the core of the problem: given a sequence of images $I_{1:K} = \{I_1, I_2, \ldots, I_K\}$ and a definition of our "target" (the cell model (4)), we need to associate the cells over time. Denote by $F_{1:K}$ a possible hypothesis of the $K$-frame association problem,

$$F_{1:K} = \{f_1, f_2, \ldots, f_K\}, \tag{10}$$

where $f_k$ is a parametric representation of frame $k$. In the case of HSCs, $f_k$ is defined as

$$f_k = \{(l_{k,j}, z_{k,j}, r_{k,j}, s_{k,j}),\ 1 \le j \le M_k\}, \tag{11}$$

where $l_{k,j}$ is the cell parent label, $z_{k,j}$ is the cell coordinate $(x_c, y_c)$, $r_{k,j}$ is the radius, $s_{k,j}$ is the cell age corresponding to cell $j$, and $M_k$ is the number of cells in frame $k$. The parent label $i = l_{k,j}$ associates cell $j$ in frame $k$ to parent cell $i$ in the previous frame. The goal is to solve the spatiotemporal cell segmentation-association problem of Figure 5: we wish to estimate $F_{1:K}$ given the image sequence $I_{1:K}$ and given an initialization $f_0$ in frame zero.

### 3.1. MAP estimation

The proposed solution to the association problem is the maximum a posteriori estimation of $F_{1:K}$:

$$\hat{F}_{1:K} = \arg\max_{F_{1:K}} P(F_{1:K} \mid I_{1:K}, f_0). \tag{12}$$

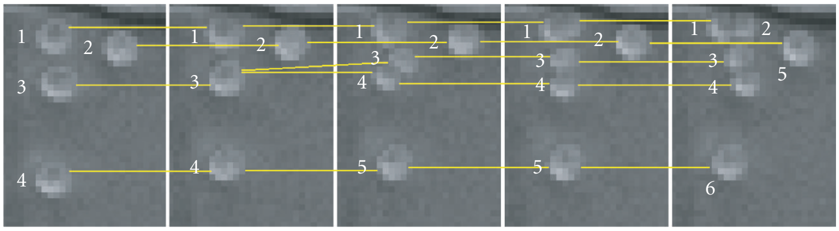

FIGURE 5: An illustration of cell association over time with numeric labels.

From Bayes' rule,

$$P(F_{1:K}, I_{1:K}, f_0) = P(F_{1:K} \mid I_{1:K}, f_0) P(I_{1:K}, f_0) = P(I_{1:K} \mid F_{1:K}, f_0) P(F_{1:K}, f_0). \tag{13}$$

As $P(I_{1:K}, f_0)$ is fixed, $\widehat{F}_{1:K}$ does not depend on it, thus

$$P(F_{1:K} \mid I_{1:K}, f_0) \propto P(I_{1:K} \mid F_{1:K}, f_0) P(F_{1:K}, f_0). \tag{14}$$

At the same time $P(f_0)$ is fixed

$$P(F_{1:K}, f_0) = P(F_{1:K} \mid f_0) P(f_0) \propto P(F_{1:K} \mid f_0). \tag{15}$$

So we conclude that

$$\widehat{F}_{1:K} = \arg\max_{F_{1:K}} P(I_{1:K} \mid F_{0:K}) \cdot P(F_{1:K} \mid f_0). \tag{16}$$

Since $F_{1:K} = \{f_1, f_2, \dots, f_K\}$, the solution to (16) is realized, in principle, by examining and evaluating all possible cell parameterizations and associations. In virtually all tracking problems of this kind the problem is made tractable by searching over a limited number of hypotheses

$$\{F^h_{1:K} \mid h = 1, 2, \dots\} \tag{17}$$

such that we find the best member of this set

$$\widehat{F}_{1:K} = F^{\widehat{h}}_{1:K}, \quad \text{where } \widehat{h} = \arg\max_h P(I_{1:K} \mid F^h_{0:K}) \cdot P(F^h_{1:K} \mid f_0). \tag{18}$$

The original, optimal solution is found if it is included among the hypotheses, that is, if

$$\arg\max_{F_{1:K}} P(F_{1:K} \mid I_{1:K}, f_0) \in \{F^h_{1:K}\}. \tag{19}$$

The key, here, to efficiency is to minimize the number of hypothesis; the key to quality of estimation is finding the most likely hypothesis. As these goals are in opposition, we are left with a complexity/quality tradeoff.

### 3.2. Evaluation of $P(I_{1:K} \mid F^h_{1:K})$

The proposed cell model $P(x_c, y_c, r \mid I_k) = P(z_c, r \mid I_k)$ from (4) evaluates the likelihood of a single cell, given an image. To solve the MAP problem we need to compute $P(I_{1:K} \mid F_{1:K})$, the likelihood of a given image sequence as a function of a specified cell parametrization and association. Since $f_k$ provides a complete paramerized description of $I_k$, conditioned on $F_{1:k}$, $I_{1:k}$ is Markov:

$$P(I_{1:K} \mid F_{1:K}) = \prod_{k \in [1,K]} P(I_k \mid f_k). \tag{20}$$

The cell model (4) describes only the likelihood of a single cell; it says nothing about groups of cells, nor does it provide any kind of prior on $z_c$ or $r$. Fortunately these latter aspects are straightforward.

(1) As the cells may be located anywhere with no prior bias, $z_c$ is uniformly distributed over the image.

(2) We empirically define a size range $r \in [2, 4]$ pixels, within which the cell radius is uniformly distributed.

(3) Any hypothesis which has cells violating the minimum required separation between cells is assigned a probability of zero.

It follows, then, that as long as zero-probability hypotheses are not created, then all remaining hypotheses $\{f^h_k\}$ are equally likely a priori. Because $P(I_k)$ is fixed, and moreover because all valid hypotheses are equally likely, such that $P(z_c, r)$ is constant, we can conclude

$$P(I_k \mid z_c, r) \propto P(z_c, r \mid I_k), \tag{21}$$

implying that the evaluation of $P(I_k \mid z_c, r)$ can follow from evaluating $P(z_c, r \mid I_k)$. The proposed parametric cell model can be applied to each image frame $I$; a two-dimensional probability map is generated, and hypothesised cells are located at local maxima of this map. Although our cell model allows this probability map to be computed as a function of $r$ for each $r \in [r_a, r_b]$, we have found that a value of $r_\circ = 2$ functions robustly. The locations of local maxima in $P(z \mid I, r_\circ)$ may either be used in the generation of hypotheses from $I$, or in computing $P(I \mid f)$, to assess an asserted cell arrangement.

First, to generate possible measurement hypotheses from $I$, find the spatial local maxima of $P(z)$ and keep only those maxima such that the likelihood of the $i$th maximum $P(z_{k,i} \mid I_k, r_\circ) > \tau$. Choosing $T$ values of $\tau$ thus generates $T$ sets of maxima, each a hypothesised measurement set for frame $k$,

$$z^\tau_k = \{z_{k,i} \mid P(z_{k,i} \mid I_k, r_\circ) > \tau,\ \tau \in [\tau_1, \tau_2]\}. \tag{22}$$

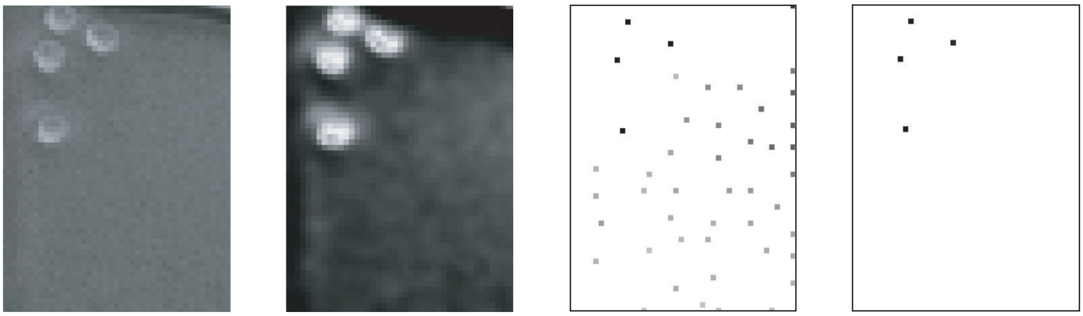

(a) (b) (c) (d)

Figure 6: (a) Microscope image $I$. (b) Probability map $P(z \mid I, r_\circ)$ obtained by applying cell model (4). (c) Local maxima of $P(z \mid I, r_\circ)$. (d) Thresholding the local maxima map.

We have found that a fixed threshold $\tau = 0.65$ works effectively for the HSC data set being considered here however, in general multiple $\tau$ would be used, leading to multiple measurement hypotheses.

Second, to compute $P(I \mid f)$ as depicted in Figure 7 the cells in $f$ are divided into two sets:

(1) $f_M$: those cells in $f$ which are located within $\delta D$ of a maximum;
(2) $f_{\overline{M}}$: those cells in $f$ which are not within $\delta D$ of a maximum.

A third set contains the unmatched maxima:

(3) $\overline{f}_M$: those maxima which are not within $\delta D$ of any point in $f$.

$f_M$ contains the successful matches, $f_{\overline{M}}$ and $\overline{f}_M$ the failed ones. The fit between $I_k$ and $f_k^h$ is thus quantified as

$$P(I_k \mid f_k^h) = \left[\prod_{j \in f_M} P(z_{k,j} \mid I_k, r_\circ)\right] \cdot \left[\prod_{i \in \overline{f}_M} \left(1 - P(z_{k,i}^\tau \mid I_k, r_\circ)\right)\right] \cdot \left[\prod_{j \in f_{\overline{M}}} P(z_{k,j} \mid I_k, r_\circ)\right], \tag{23}$$

where $P(z_{k,j} \mid I_k, r_\circ)$ is the probability of the location of the $j$th cell in the state $f_k^h$ for frame $k$, and $P(z_{k,i}^\tau \mid I_k, r_\circ)$ is the probability of the $i$th maximum in frame $k$. As depicted in Figure 7, $P(I_k \mid f_k^h)$ is evaluated by applying (23).

### 3.3. Evaluation of $P(F_{1:K}^h \mid f_0)$

The second part of (18) is the evaluation of association hypotheses $\{F_{1:K}^h\}$. To track the HSCs over time, detected cells in the measurement hypothesis of the current frame $z_k^\tau$ must be associated to the most probable element in the previous frame.

Considering that for each image frame $k$, we associate cell features from $(k-1)$ only, Markovianity can be asserted on $F_{1:K}$ such that

$$P(F_{1:K} \mid f_0) = \prod_{k \in [1,K]} P(f_k \mid f_{k-1}), \tag{24}$$

where we recall that $f_k$ is the set of cell properties in frame $k$. The cell age $s_{k,j}$ is updated as

$$s_{k,j} = \begin{cases} 1 & \text{if } \exists m \text{ such that, } l_{k,m} = l_{k,j} \\ & \text{(i.e., cell split),} \\ \left(s_{(k-1),l_{k,j}}\right) + 1 & \text{otherwise.} \end{cases} \tag{25}$$

Each cell in $f_k$ must belong to one of the following sets.

Unassociated: $N = \{j \mid l_{k,j} = 0\}$.
Split: $S = \{j \mid l_{k,j} = l_{k,m} \text{ for } m \neq j\}$.
Regular: $R = \{j \mid j \notin \{N \cup S\}\}$.

In contrast with joint probabilistic data association (JPDA) [26–28] in which new tracks can not be initiated, our proposed method initiates new tracks for divided cells, therefore the following constraints are considered:

(i) each measurement must originate from cell or clutter;
(ii) each measurement can be associated to one cell;
(iii) up to two measurements in frame $k$ can be associated to the same cell in frame $k-1$.

Asserting Markovianity we evaluate $P(f_k^h \mid f_{k-1}^h)$ in thef rest of this section. The association problem is resolved frame by frame by selecting the hypothesis with the maximum joint association probability. In this way the measurement hypothesis $z_k^\tau$ for frame $k$ and association hypothesis $f_{k-1}^h$ from the previous frame are used to generate hypotheses $f_k^h$. Therefore we have

$$P(f_k^h \mid f_{k-1}^h) = P(f_k^h \mid z_{1:k}^\tau, f_{k-1}^h) = P(f_k^h \mid z_k^\tau, f_{k-1}^h). \tag{26}$$

The filter step is

$$P(f_k^h \mid f_{k-1}^h) = P(f_k^h \mid z_{1:k}^\tau, f_{k-1}^h) = \frac{P(f_k^h \mid z_{1:k-1}^\tau) \cdot P(z_k^\tau \mid f_k^h)}{P(z_k^\tau \mid z_{1:k-1}^\tau)}, \tag{27}$$

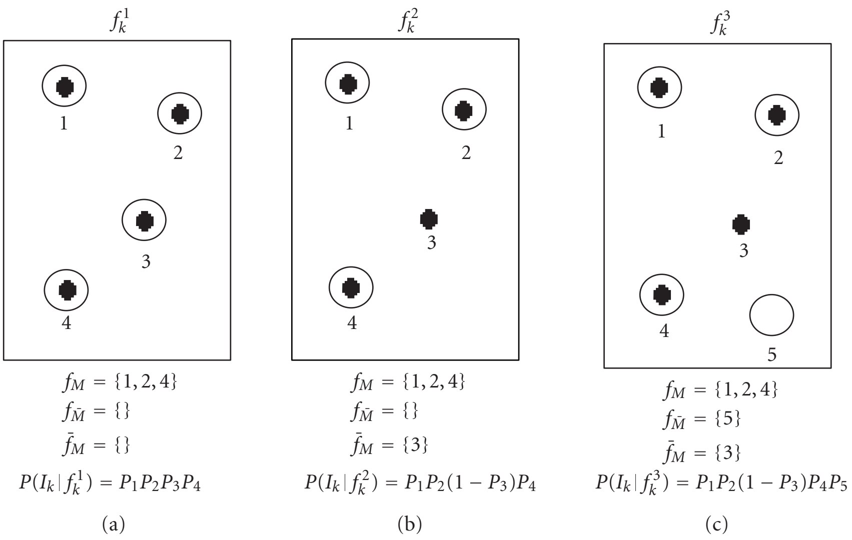

FIGURE 7: Having 4 maxima (solid dots), and 3 hypothesized sets of cells (large circles), for each hypothesis $f_M$, $f_{\overline{M}}$, and $\overline{f}_M$ are illustrated and then $P(I_k \mid f_k^h)$ is evaluated.

$P(z_k^\tau \mid z_{1:k-1}^\tau)$ is fixed and we have

$$P(f_k^h \mid f_{k-1}^h) = \lambda_k \cdot P(f_k^h \mid z_{1:k-1}^\tau) \cdot P(z_k^\tau \mid f_k^h), \tag{28}$$

where $\lambda_k$ is a normalization constant. The first term of (28), $P(f_k^h \mid z_{1:k-1}^\tau)$, is a prediction step which is illustrated as follows. Because of the nonlinear and non-Gaussian nature of both measurements and dynamics, in contrast with JPDA, the Kalman filter is not considered for the prediction step. The prediction step in the proposed method is

$$\begin{aligned} P(f_k^h \mid z_{1:k-1}^\tau) &= \int P(f_k^h \mid f_{k-1}^h) P(f_{k-1}^h \mid z_{1:k-1}^\tau) df_{k-1}^h \\ &= \left[\prod_{j\in R\cup S} P_{\text{vel}}(z_{k,j}, z_{k-1,l_{k,j}}^\tau)\right] \\ &\quad \cdot \left[\prod_{j\in S} P_{\text{state}}(s_{k-1,j})\right]. \end{aligned} \tag{29}$$

The former term $P_{\text{vel}}$ is a nonlinear term to predict the location of the hypothetical cell $j$ in frame $k$ based on its dynamics and its location in frame $k-1$. The motion will be cell-type specific, and may further be influenced by environmental factors, chemical gradients, and so forth. In our context there are no deliberate experimental biases, and a Gaussian random walk was found to well-approximate hand-tracked cell motion. The latter term $P_{\text{state}}$ predicts the likelihood of cell division in frame $k$. An age penalty such that cell division cannot happen below some age; this minimum age is cell-type specific and is asserted from biological experience.

The second term of (28), $P(z_k^\tau \mid f_k^h)$, is the likelihood of measurement $z_k^\tau$ given hypothesis $f_k^h$ and is given by

$$\begin{aligned} P(z_k^\tau \mid f_k^h) &= \left[\prod_{j\in R\cup S} N(v_{k,j}, 0, S_{k,j})\right] \\ &\quad \cdot \left[P_{\text{sep}}(f_k^h)\right] \cdot \left[\prod_{j\in N} P_{\text{una}}\right], \end{aligned} \tag{30}$$

where $v_{k,j} = z_{k,i}^\tau - \hat{z}_{k,j}$ is an innovation term so that the $i$th measurement is within $\delta D$ of the $j$th hypothesised cell location in frame $k$. $P_{\text{una}}$ is a penalty on the association of unassociated cells, and $P_{\text{sep}}$ is the probability of separation distance of a measurement pair. As we can see in the proposed method, the likelihood of measurement $z_{k,j}$ is penalized by the unlikely events such as minimum separation distance and unassociated cells.

### 4. EXPERIMENTAL RESULTS

We begin by evaluating the proposed cell model. The model generates cell hypotheses, as illustrated in Figure 6, where candidate cells are found as local maxima in $P(x_c, y_c, r) = P(z_c, r)$. Because of the availability of hand-labelled ground-truth data, a good assessment of cell detection is possible. The only unknown parameter in cell detection is $\tau$, the probability threshold in declaring a cell present. Figure 8 shows the probability of false alarm and missed detection as a function on the chosen threshold $\tau_h$ over a sequence of HSC phase contrast microscope images. It is clear that a threshold yielding acceptably low failures of both types is $\tau = 0.65$.

Next, Figures 9 and 10 test the number of detected cells (using $\tau = 0.65$) and the detected spatial locations with manual ground truth. The greatest probability of misdetection

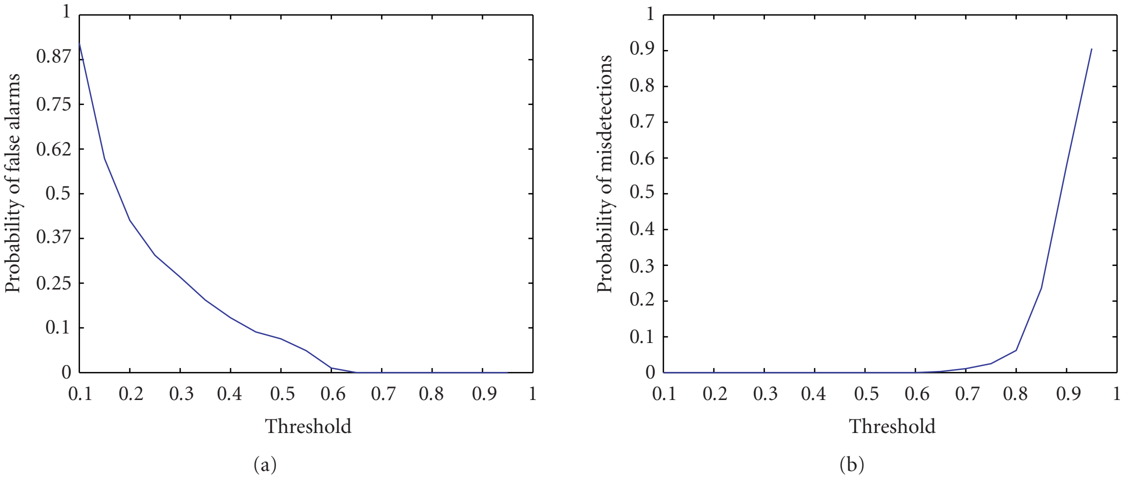

FIGURE 8: (a) Variation of the probability of false alarm as a function of the threshold $\tau_h$. (b) Variation of the probability of missed detection as a function of the threshold $\tau_h$.

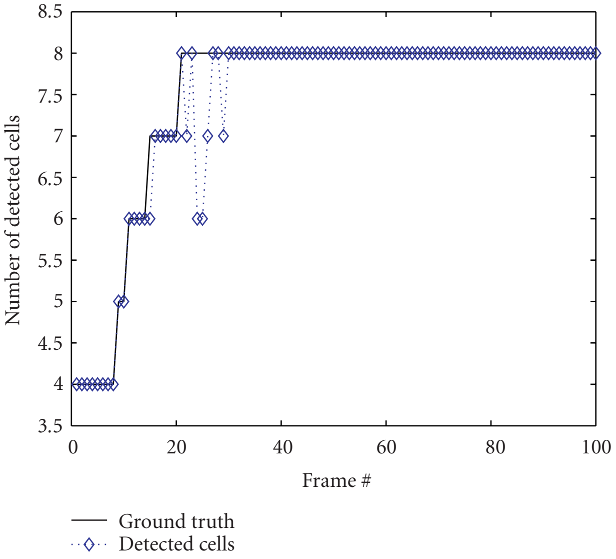

FIGURE 9: A comparison of ground truth (solid line) and the detected cells on the basis of the proposed cell model (dashed line).

occurs during division where a mature cell gives rise to two new cells. During division, notable between frames 9 to 29, the cells are inconsistent with a circular shape with a fixed radius. However, the divided cells are recognized as soon as the division process is completed, which takes at most five frames. As it can be seen in Figure 10, the maximum cell center spatial error is 1.8 pixels per cell. Considering the fact that manual ground truth is prone to error, the results obtained by the proposed method are very promising.

Results obtained by applying the proposed probabilistic cell tracking method are depicted in Figure 11. Cell centers are detected by applying the cell model (4), locating the local maxima in the probability map, and thresholding the local maxima map. Finally cell centers are associated using the proposed tracking method. Color coding is used to highlight associated cell centers such that different colors show the association of cell centers over time. It takes about 0.5 seconds for a well to be segmented and associated in the current stage using a *Pentium* 4 running at 1.6 Ghz.

As can be observed from Figure 11, by applying our probabilistic model-based tracking to HSC image sequence, it is able to identify and associate both nondividing and dividing cell centers correctly. However there are some cases, such as having a large number of proximate young divided cells or a few number of nearby dividing mature cells, in which some of the hypotheses have very similar probability, therefore deriving the best hypothesis for such a frame is very difficult and prone to error. Employing a more robust Bayesian approach will resolve those ambiguous situations over time by the further integration of information of neighboring frames, maintaining several hypotheses, and selecting the most likely one over the subsequent images.

### 5. CONCLUSIONS

Image cytometry is a practical approach to measure and extract cell properties from large volumes of microscopic cell images. As an important application of image cytometry, this paper presents a probabilistic model-based cell tracking method to locate and associate HSCs in phase contrast microscopic images.

Our statistical cell model, which is constructed after carefully observing HSCs in typical image sequences, captures the key properties of these cells. The close match between the model and imaged HSCs allowed for threshold selection yielding very low false alarm or missed detection. Cells in isolation are detected well; recently split cells provide a proper fit to the model and rely on association to resolve ambiguities.

Cell association is accomplished based on the proposed joint association method. As it can be observed in Figure 2 the cell dynamics can be well approximated by a random

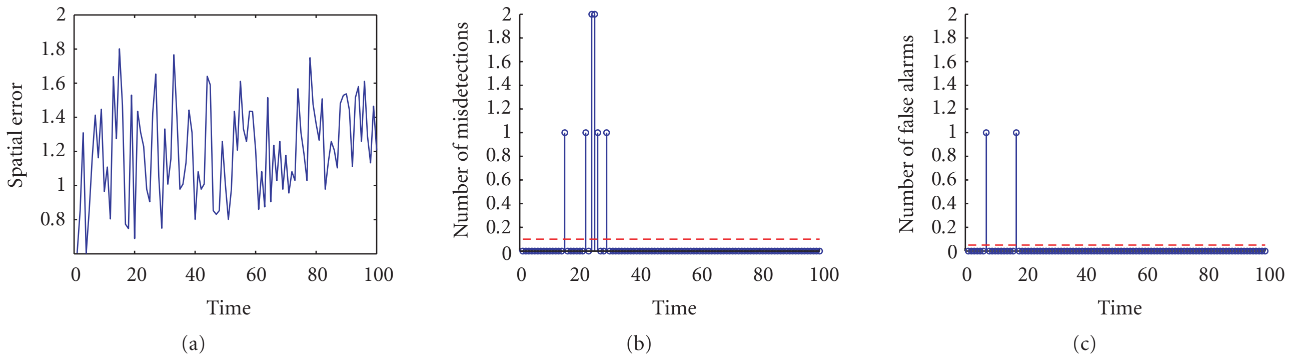

Figure 10: (a) RMS spatial error, the distance between detected cell centers and ground truth. (b) The location of missed detections with the superimposed average missed detection probability (0.0107). (c) The location of false alarms with the superimposed average false alarm probability (0.0027).

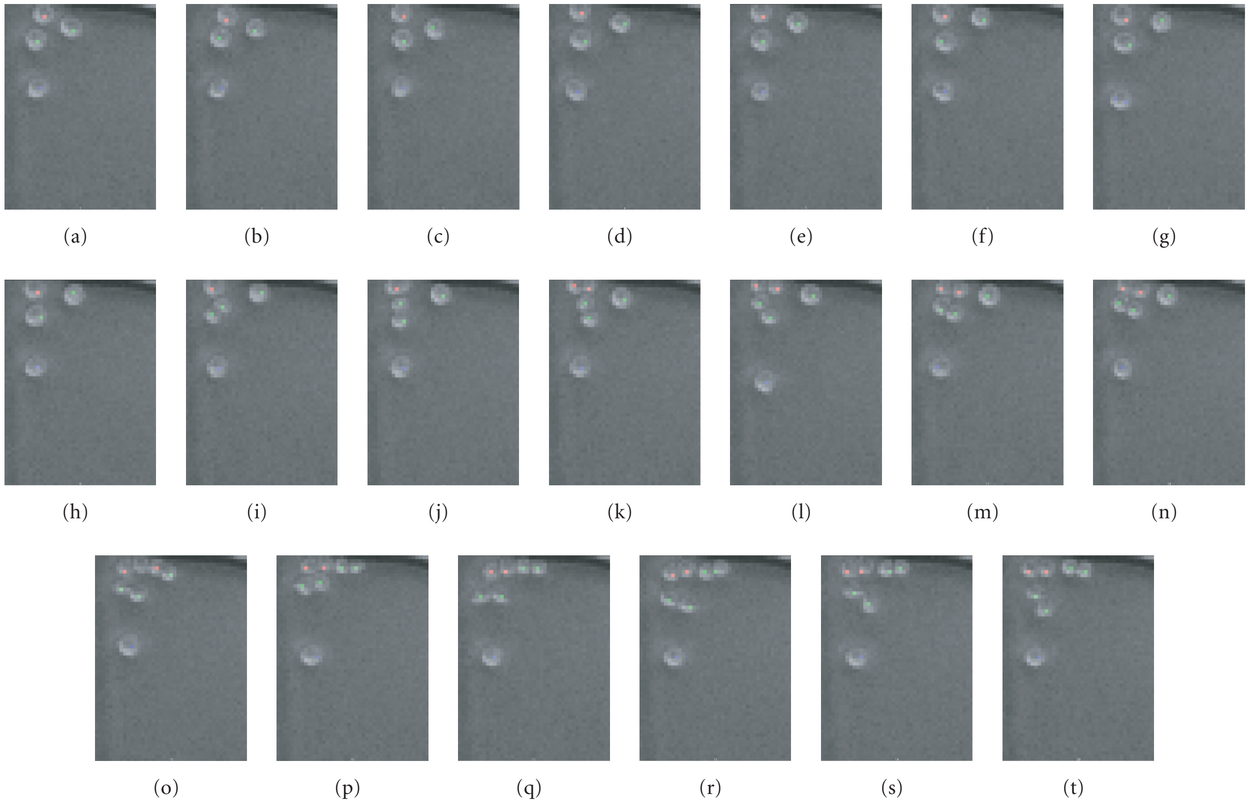

Figure 11: Detection and association of cell centers obtained by applying the proposed model-based tracking method. Results are superimposed on the original HSC images and each color shows a different cell track over time (frames 1–20).

walk as it has been considered in the proposed method to model the cell motion. It can be seen from the previous section that such a probabilistic model-based cell tracking method produces promising results and is able to identify and associate both dividing and nondividing cell centers correctly. However, there are some cases, such as a few proximate dividing cells or large number of nearby cells, in which the proposed method may be inaccurate. To resolve association ambiguities in such cases and to make the method more robust to noise and clutter, future work will be conducted to extend the proposed method by integrating the information over multiple neighboring frames.

Future work will also include improving the cell model to more accurately reflect unique properties of the cells under different conditions. Moreover further work is required to better preprocess the images with background subtraction to improve homogeneity and eliminate camera artifacts. There is also considerable interest in designing a parametric cell model with additional degrees of freedom to generate lineage trees in which cells can be characterized by richer features so that cell properties can be more reliably extracted.

### ACKNOWLEDGMENTS

This research has been funded by Natural Science and Engineering Research Council of Canada (NSERC). This research has been also performed in close cooperation with Chemical Engineering Department (CE) of the University of Waterloo (UW) and Terry Fox Laboratory (TFL) of the University of British Columbia (UBC). We would like to thank professor Connie Eaves and Mr. Brad Dykstra from TFL-UBC and researchers in Cancer Research Laboratory (CRL) of UBC for providing cell samples for this research and valuable inputs on biological properties of HSCs.

### REFERENCES

[1] P. M. Banks, J. Chan, M. L. Cleary, et al., "Mantle cell lymphoma: a proposal for unification of morphologic, immunologic, and molecular data," *American Journal of Surgical Pathology*, vol. 16, no. 7, pp. 637–640, 1992.

[2] I. Baumann, R. Nenninger, H. Harms, et al., "Image analysis detects lineage-specific morphologic markers in leukemic blast cells," *American Journal of Clinical Pathology*, vol. 105, no. 1, pp. 23–30, 1996.

[3] E. Campo and E. S. Jaffe, "Mantle cell lymphoma. Accurate diagnosis yields new clinical insights," *Archives of Pathology and Laboratory Medicine*, vol. 120, no. 1, pp. 12–14, 1996.

[4] J. K.C. Chan, P. M. Banks, M. L. Cleary, et al., "A revised European-American classification of lymphoid neoplasms proposed by the International Lymphoma Study Group: a summary version," *American Journal of Clinical Pathology*, vol. 103, no. 5, pp. 543–560, 1995.

[5] Y. Cheng, "Mean shift, mode seeking, and clustering," *IEEE Transactions on Pattern Analysis and Machine Intelligence*, vol. 17, no. 8, pp. 790–799, 1995.

[6] T. Markiewicz, S. Osowski, L. Moszczyński, and R. Sałat1, "Myelogenous leukemia cell image preprocessing for feature generation," in *Proceedings of 5th International Workshop on Computational Problems of Electrical Engineering*, pp. 70–73, Jazłoweic, Ukraina, August 2003.

[7] V. Meas-Yedid, F. Cloppet, A. Roumier, A. Alcover, J.-C. Olivo-Marin, and G. Stamon, "Quantitative microscopic image analysis by active contours," in *Proceedings of Vision Interface Annual Conference - Medical Applications (VI '01)*, pp. 277–284, Ottawa, Ontario, Canada, June 2001.

[8] K. Wu, D. Gauthier, and M. D. Levine, "Live cell image segmentation," *IEEE Transactions on Biomedical Engineering*, vol. 42, no. 1, pp. 1–12, 1995.

[9] D. A. Forsyth and J. Ponce, *Computer Vision - A Modern Approach*, Prentice-Hall, Upper Saddle River, NJ, USA, 2003.

[10] A. Blake and M. Isard, *Active Contours*, Springer, New York, NY, USA, 1997.

[11] D. Marr, *Vision: A Computational Investigation into the Human Representation and Processing of Visual Information*, W. H. Freeman, San Francisco, Calif, USA, 1982.

[12] S. J. Osher and R. P. Fedkiw, *Level Set Methods and Dynamic Implicit Surfaces*, Springer, New York, NY, USA, 2002.

[13] J. A. Sethian, *Level Set Methods and Fast Marching Methods Evolving Interfaces in Computational Geometry, Fluid Mechanics, Computer Vision and Materials Science*, Cambridge University Press, Cambridge, UK, 1999.

[14] L. Shapiro and G. C. Stockman, *Computer Vision*, Prentice-Hall, Upper Saddle River, NJ, USA, 2001.

[15] E. Trucco and A. Verri, *Introductory Techniques for 3D Computer Vision*, Prentice-Hall, Upper Saddle River, NJ, USA, 1998.

[16] C. Rasmussen and G. D. Hager, "Probabilistic data association methods for tracking complex visual objects," *IEEE Transactions on Pattern Analysis and Machine Intelligence*, vol. 23, no. 6, pp. 560–576, 2001.

[17] E. Jervis, "Living cell tracking," http://cape.uwaterloo.ca/~ericjj.

[18] D. Comaniciu and P. Meer, "Cell image segmentation for diagnostic pathology," in *Advanced Algorithmic Approaches to Medical Image Segmentation: State-of-the-Art Applications in Cardiology, Neurology, Mammography and Pathology*, pp. 541–558, Springer, New York, NY, USA, 2001.

[19] D. Demandolx and J. Davoust, "Multiparameter image cytometry: from confocal micrographs to subcellular fluorograms," *Bioimaging*, vol. 5, no. 3, pp. 159–169, 1997.

[20] G. I. Nistor, M. O. Totoiu, N. Haque, M. K. Carpenter, and H. S. Keirstead, "Human embryonic stem cells differentiate into oligodendrocytes in high purity and myelinate after spinal cord transplantation," *GLIA*, vol. 49, no. 3, pp. 385–396, 2005.

[21] W. Bieniecki, "Oversegmentation avoidance in watershed-based algorithms for color images," in *Proceedings of IEEE International Conference on Modern Problems of Radio Engineering, Telecommunications and Computer Science (TCSET '04)*, pp. 169–172, Lviv-Slavsko, Ukraine, February 2004.

[22] D. Anoraganingrum, "Cell segmentation with median filter and mathematical morphology operation," in *Proceedings of IEEE International Conference on Image Analysis and Processing (ICIAP '99)*, pp. 1043–1046, Venice, Italy, September 1999.

[23] H.-S. Wu, J. Barba, and J. Gil, "Region growing segmentation of textured cell images," *Electronics Letters*, vol. 32, no. 12, pp. 1084–1085, 1996.

[24] N. Otsu, "A threshold selection method from gray-level histograms," *IEEE Transactions on Systems, Man and Cybernetics*, vol. 9, no. 1, pp. 62–66, 1979.

[25] C. Glasbey, "An analysis of histogram-based thresholding algorithm," *Graphical Models and Image Processing*, vol. 55, no. 6, pp. 532–537, 1993.

[26] Y. Bar-Shalom and K. Birmiwal, "Consistency and robustness of PDAF for target tracking in cluttered environments," *Automatica*, vol. 19, no. 4, pp. 431–437, 1983.

[27] S. Colegrove, A. Davis, and J. Ayliffe, "Track initiation and nearest neighbours incorporated into probabilistic data association," *Journal of Electrical and Electronics Engineering*, vol. 6, no. 3, pp. 191–198, 1986.

[28] Y. Bar-Shalom and T. E. Fortmann, *Tracking and Data Association*, Academic-Press, San Diego, Calif, USA, 1988.

---

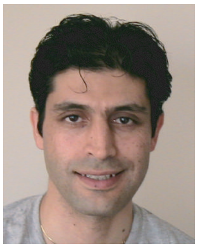

**Nezamoddin N. Kachouie** received his B.S. degree in computer engineering from the Isfahan University of Technology (IUT). He received his M.S. degree in electrical and computer engineering from the Ryerson University, Canada, in 2004 and since then he has been with the Department of Systems Design Engineering at the University of Waterloo, Canada, completing his Ph.D. degree in the field of biomedical engineering.

He is interested in deformable models and Bayesian framework for biomedical applications of multiple target tracking. His current research focuses on segmentation and tracking of stem cells in biomedical multicellular videos by designing a model-based tracking system. This tracking system will be used to study stem cell behavior which is of crucial importance in drug and disease research. His research interests also include statistical signal processing, content-based image retrieval, image registration, object recognition, spatiotemporal image analysis, image denoising, optimization, and classification.

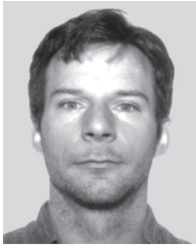

**Paul Fieguth** received the B.S. degree from the University of Waterloo, Ontario, Canada, in 1991 and the Ph.D. degree from the Massachusetts Institute of Technology, Cambridge, in 1995, both degrees in electrical engineering. He joined the Faculty at the University of Waterloo in 1996, where he is currently an Associate Professor in systems design engineering. He has held visiting appointments at the Cambridge Research Laboratory, at Oxford University, and the Rutherford Appleton Laboratory in England, and at INRIA/Sophia in France, with postdoctoral positions in computer science at the University of Toronto and in information and decision systems at MIT. His research interests include statistical signal and image processing, hierarchical algorithms, data fusion, and the interdisciplinary applications of such methods, particularly to remote sensing.

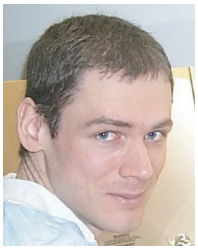

**John Ramunas** Since his graduation with a B.S. degree in biochemistry from the University of Waterloo in 2001 he has been working in the lab. of Eric Jervis developing technologies for studying and controlling cells, for example, arrays of glass-bottom microwells that allow high-quality long-term imaging of cells, and software for manually scoring characteristics of movies of cells cultured in these microwells. This scoring process is a bottleneck in the experiment pipeline so it is with great enthusiasm that he welcomes the advances offered by the work of Nezamoddin and Paul. He serves as a liason to help integrate these advances into the existing software and test them in an iterative cycle involving actual ongoing experiments. He will begin his Ph.D. at Stanford in 2006 after which other members of the Jervis lab. will continue the development cycle with the Fieguth lab.

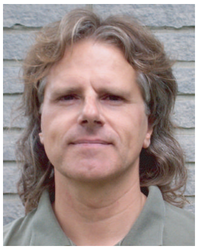

**Eric Jervis** received his undergraduate and graduate degrees in chemical and biological engineering from the University of British Columbia. Upon completing his Ph.D. in 1998, he took a position in chemical engineering at the University of Waterloo and was subsequently granted tenure and promoted to the rank of Associate Professor in 2004. He is currently developing three lines of research that provide leading-edge opportunities for student training: (a) development of image-based lineage tracking tools for examining cell division symmetry and cell potential maintenance in stem cell cultures—a "lineage informatics" approach to cell culture analysis; (b) development of multiphoton-based methodologies for photoactivation of caged molecules for drug delivery; and (c) development of improved high-density bioreactor protocols with optical bioprocess monitoring instrumentation. These research themes are united by the application of high-resolution imaging and photonics-based analytical techniques. He is a Member of the Canadian Stem Cell National Centers of Excellence.

